# The Latent Perception of Pregnancy

**DOI:** 10.3389/fpsyg.2022.589911

**Published:** 2022-03-24

**Authors:** Leah Borovoi, Shoshana Shiloh, Lailah Alidu, Ivo Vlaev

**Affiliations:** ^1^Department of Education and Psychology, The Open University of Israel, Raanana, Israel; ^2^School of Psychological Sciences, Tel Aviv University, Tel Aviv, Israel; ^3^Institute of Applied Health Research, University of Birmingham, Birmingham, United Kingdom; ^4^Warwick Business School, University of Warwick, Coventry, United Kingdom

**Keywords:** pregnancy, obstetrics, health beliefs, risk perception, women—health and hygiene

## Abstract

**Background:**

The main purpose of this study was to describe the latent structure of pregnancy perception by investigating the role of risks and medical examinations in pregnancy perception across the sexes and pregnancy status.

**Methods:**

Study 1 developed a questionnaire based on the responses of 29 young adults on their perception of pregnancy. Study 2 consisted of distributing the questionnaire among 290 participants (mean age 29.3; standard deviation = 7.5).

**Results:**

The statistical clustering analysis revealed three major clusters of pregnancy perceptions: “evaluative,” “physio-medical,” and “future considerations,” each of them encompassing several meaningful sub-clusters. This structure of pregnancy perceptions supports Beck and Beck-Gernsheim’s modernization approach. Negative emotions toward pregnancy were related to social cognitions, whereas thoughts about risks were included in the medical sub-cluster. After reliability analyses, comparisons of scale scores revealed that women experienced more positive emotions, thought more about physical symptoms and about future issues compared to men (evolutionary explanation was offered).

**Conclusion:**

Pregnant participants felt less ambivalence toward pregnancy, thought more about risks and medical examinations and less about parents’ duties than non-pregnant participants.

## Introduction

Pregnancy is a major life event, during which women experience a wide range of psychological, physical, emotional, and personal changes. One pregnancy differs from another, and a lot of uncertainty accompanies each pregnancy. Whereas in the 19th-century songs and poems often linked pregnancy and childbirth with women’s nature, idealizing the feelings and giving them a romantic aura, today’s pregnancy is a medicalized topic, overloaded by instructions and educational books telling the mother-to-be how to behave in order not to harm the fetus (take folic acid, refrain from smoking, alcohol, etc.; Beck and Beck-Gernsheim, 1995). What has been the most natural thing in the world has become highly complicated. Obstetricians argue that the perception of pregnancy and birth has changed from a basically natural event to a life-threatening situation, unless contrary evidence is given (Enkin, 1994; Enkin et al., 2006; Downe, 2010; Robinson et al., 2011). It is common knowledge that pregnancy’s risk to the mother’s life has dropped progressively during the last 70 years. Most women, doctors, and midwives will never see a woman die of giving birth. On the other hand, the unborn baby became the subject of great concern. Separate and distinct from the mother, it is perceived as under the guardianship and protection of the obstetrician. The development of new diagnostic tools, such as ultrasound or cardiography, enables direct observation of the fetus’ condition and intervention (e.g., caesarean section) if the fetus appears to be in trouble. Revealed congenital anomalies [e.g., congenital bilateral urinary tract obstruction (UTO), congenital diaphragmatic hernia (CDH), and congenital pulmonary anomalies] can be corrected by surgery before birth in certain cases. Amniocentesis and other invasive prenatal examinations for abnormalities offer an opportunity to terminate pregnancy if abnormalities are detected. Women are encouraged to take all kinds of precautions to protect their baby and to take as many prenatal examinations as possible. These developments are assumed to have changed the phenomenological concept of pregnancy in developed countries, by re-focusing perceptions on fetal risks and prenatal diagnostic procedures. One aim of the present study was to examine how prenatal diagnostic procedures and fetal risks have been incorporated within the contents and structure of modern pregnancy perceptions.

### Risk Perception in Pregnancy

Much of the psychological research on pregnancy is dedicated to prenatal anxiety, maternal stress, and depression (Levin and DeFrank, 1988; Rini et al., 1991; Diego et al., 2004; Field et al., 2008; Räikkönen et al., 2011; Hollands et al., 2016). Research on risk perceptions during pregnancy and its effects on uptake of prenatal diagnosis have been growing over the years. Physicians and parents use more prenatal testing if their attitudes toward prenatal testing and termination of pregnancy are more positive (Sagi et al., 1992). Presenting prenatal testing as routine (which does not conform to “informed consent” demands) results in higher rates of uptake (Marteau et al., 1988; Dormandy et al., 2007; van den Heuvel et al., 2010). Genetic risk perceptions and prenatal diagnostic decisions were found related to having an affected child or any personal experience with the disorder, women’s age, desire to have children, and the objective level of genetic risk (Shiloh, 1996; Shiloh et al., 2002, 2006). In sum, the concept of “risk” clearly dominates today’s discourse about pregnancy, sometimes conceptualized as “the culture of extreme risk aversion” (Shiloh et al., 2001; Ballantyne et al., 2016).

It is important to note that while medical practitioners and genetic counselors refer to risk as the probability of occurrence of negative outcome, parents and genetic counselees attach more global and personal meaning to risk, sometimes interpreted as the severity of the disorder (Shiloh, 1996; Shiloh et al., 2001; Ballantyne et al., 2016). Parents’ understanding of risk often depends on values, education, and cultural background (Handwerker, 1994; Lupton, 1999a,b, 2012; Donovan, 2006; Kukla, 2010). Differences in the interpretations of risk were demonstrated as a major obstacle to effective communication between doctors and patients. Furthermore, when addressing pregnancy risks it is sometimes unclear what the focus risk event is: for example, the risk for giving birth to an abnormal baby? for miscarriage? for delivery complications or mother’s health? We believe that better understanding of perceived risk during pregnancy and its position within the cognitive schema of pregnancy may contribute to improvements in communication with prospective parents.

### Gender Differences

Evolutionary psychology predicts that gender differences in behavior and perception occur in domains where the genders have faced different adaptive problems (Buss and Schmitt, 2011). In humans and other mammals, male investment in reproduction tends to be smaller than female investment (e.g., 9-month gestation, lactation, and protection). Evolutionary theory predicts that women who selected men who were able to invest resources in them and their offspring had a tremendous advantage in survival and reproductive currencies compared with women who were indifferent to the investment capabilities of the man with whom they chose to mate (Buss, 1995, 2000). These evolutionary differences are expected to be partly expressed in how men and women perceive pregnancy.

### Comparisons Between Pregnant and Non-pregnant Participants

The perception of pregnancy is also believed to depend on whether pregnancy is hypothetical or presently experienced. According to Construal Level Theory (CLT; Liberman and Trope, 2008, 2014), individuals construct different representations of the same event depending on whether the event is taking place at present or will occur in a distant future. The theory maintains that distant future events are perceived by higher-level construals, whereas events that take place now or in the near future are perceived by low-level construals. High-level construals consist of general, abstract schemas and super-ordinate, essential, features—while low level construals are more concrete, specific, sub-ordinate, and describe incidental features of objects or events. “Why” aspects of phenomena and desirability considerations (i.e., the value of an outcome) constitute a high level construal, receiving more weight in perceiving distant future events—whereas “how” aspects of those phenomena and feasibility considerations (i.e., ease or difficulty in reaching the outcome, resources required for implementation) constitute a low level construal and receive more weight in perceiving near future events (Liberman and Trope, 2014). Applying the theory to pregnancy perception implies that those for whom pregnancy is a distant future event will perceive it by high-level features, such as its desirability, parenthood duties (e.g., passing values), and motivations to conceive. On the other hand, if pregnancy is experienced now, low-level construals would dominate, such as bodily sensations, changes in body weight and appearance, physical symptoms, and medical examinations. Psychological distance can also be expressed by the “me–not me” distinction. Therefore, we also predicted that women, who experience pregnancy personally, will perceive pregnancy by more low-level construals than men.

### The Present Study

The major purpose of this study was to understand the nature and the latent structure of the modern concept of pregnancy. Rather than using *a priori* formulations, we intended to investigate and reveal how pregnancy is perceived and experienced by ordinary people. Based on Evolutionary Theory and Temporal Construal Theory we predicted differences in pregnancy representations between men and women, participants who are pregnant and those who are not. To our knowledge, this is the first empirical study aimed to examine these questions.

## Materials and Methods

The following procedures were applied: (1) a qualitative study asking participants about their thoughts and feelings about pregnancy, without restraining their responses; (2) generation of a questionnaire based on their responses and distributing it among pregnant and non-pregnant populations; (3) analyzing the questionnaire data using a clustering analysis procedure; (4) identifying the meanings of clusters, scoring them as sub-scales of pregnancy perceptions and testing their reliabilities; and (5) comparing scale scores between men and women, participants who are pregnant and those who are not. The ethics committee of Tel Aviv University reviewed and approved the research before the start of data collection.

### Study 1: Development of the Questionnaire

We interviewed nine pregnant women recruited in a pregnancy clinic, and 20 young adults who did not intend to have a child in the near future (six men and 14 women). We asked the participants to write on a paper their responses to the following open-ended question: “Think about pregnancy. What comes into your mind?” The question was designed to elicit participants’ thoughts, emotions, and experiences without constraining their responses. Participants were encouraged to mention anything that comes to their minds while thinking about pregnancy. Participants’ responses were transcribed into 212 written “meaning units” that reflected the perceived concept of pregnancy while retaining participants’ language. Two independent researchers evaluated the sentences, eliminated redundancies, considered their importance, and agreed upon a final list of 68 statements to be included in the questionnaire (similar methodology is reported in the literature; Daughtry and Kunkel, 1993; Paulson et al., 1999). The questionnaires were administered in Hebrew, which is the native language of the authors and the respondents. The final list included items describing emotional and cognitive reactions to pregnancy.

### Study 2: Main Study

#### Participants

Two hundred and ninety subjects, mean age 29.3 (*SD* = 7.5), age (18–65) participated in the second part of this study. Distributions of demographic data are presented in Table 1. All participants were asked to fill out a research questionnaire about pregnancy voluntarily. The researcher asked each person if he or she would like to participate in the study. If he/she agreed, they were then given the questionnaire to complete in the waiting area. The questionnaire was returned to the researcher when they completed it. More than 90% of those who received the questionnaire answered it. Two hundred and thirteen participants were born in Israel, 43 were immigrants from the former Soviet Union, 27 from other countries, and seven did not report their origin. In this research the use of the word “pregnant” does not explicitly specify that the person is a woman, we mean “pregnant or having a pregnant partner.”

**Table 1:** Distributions of demographic variables.

**Pregnant (***N*** = 91)**		**Non-pregnant (***N*** = 198)**	
Men: *N* = 25	Women: *N* = 66	Men: *N* = 55	Women: *N* = 143
Age: Mean = 27.8 years, *SD* = 8.1		Age: Mean = 32.6 years, *SD* = 4.6	
Gestational age: *M* = 21.9 months, *SD* = 8.4 High risk: *N* = 21		Trying to conceive: *N* = 29 Pregnant friend/relative: *N* = 55	
Education: Mean = 15.0 years, *SD* = 2.4		Education: Mean = 15.2 years, *SD* = 2.2	
Children: *N* = 53 (58%) have children		Children: *N* = 42 (21%) have children	

#### The Pregnancy Perceptions Questionnaire

The first page of the questionnaire included an invitation to participate in a research about perception of pregnancy. It was stated that there were no right or wrong answers and that expressing genuine opinions was very helpful and important. The following pages presented 68 items generated from study 1, each describing one aspect of pregnancy perception, for example: “thoughts about birth complications.” Participants were asked to rate (on 7-point scales) the degree to which each statement matched their thoughts and feelings (1—“Never crossed my mind” to 7—“Matches my thoughts very well”). The last page of the questionnaire asked for general background information: age, gender, and years of education, birthplace, whether or not the participant or his partner are pregnant, has experienced pregnancy, has children, knows somebody close who is pregnant and intention to conceive in the future.

#### Procedure

After consenting to participate in the study, non-pregnant participants filled-out the questionnaire (pencil and paper) individually at a library in the University. Fewer than 10% did not finish filling out the questionnaire and were not included in the study. Pregnant participants were met at Sheba Medical Center and responded to the questionnaire in groups of 5–15 people. All participants were asked to think for a minute about pregnancy and fill out the questionnaire.

#### Statistical Analysis

A clustering algorithm called ADDTREE (i.e., additive similarity tree; Sattath and Tversky, 1977) was performed using a similarity matrix (Pearson’s correlations) among the questionnaire items (see Supplementary Material 3). This procedure was aimed to group the items into internally consistent clusters. ADDTREE is generally used to provide a clustering representation of a concept and represent dissimilarities among items. This method has been successfully used to analyze the structure of career-related aspects (Gati et al., 1995, 1996; Nudelman and Shiloh, 2015; Preis and Benyamini, 2017), as well as health behaviors and illness attributions (Gati et al., 1996; Shiloh et al., 2002; Nudelman and Shiloh, 2015). ADDTREE is especially attractive because it graphically represents the proximity matrix in the form of an additive or “path length” tree, in which the variables are divided into clusters and sub-clusters according to the proximity between them (based on the correlation matrix). The distance between any pair of items is represented in the clustering structure by the sum of horizontal arcs on the shortest path connecting them. Items that have common features are clustered together, while different clusters represent distinctive features. In our study, the relationship between an item and the corresponding cluster is essentially the relationship between a specific statement in the questionnaire and the notion of pregnancy represented by a set of statements included in the same cluster. The accuracy of the analysis was measured by two goodness of fit indices: Kruskal’s Stress formula, which is an index of the stability of the ADDTREE solution and ranges from zero (perfectly stable) to 1 (perfectly unstable), and *R*^2^ (the linear variance accounting for each solution). The larger the *R*^2^, the better the configuration represents the data (Gati et al., 1996; Nudelman and Shiloh, 2015).

Two independent researchers reached agreement about labeling the clusters according to their constituent items. Items that composed each sub-cluster were averaged to form sub-scale scores. The psychometric properties of the questionnaire were examined, focusing on scales’ means, standard deviations, and internal reliabilities (Cronbach’s alpha). Finally, using t-tests for independent samples, scale scores were compared between men and women, and between pregnant and non-pregnant participants.

### Patient and Public Involvement

The development of the research question and outcome measures were informed by patients’ priorities, experience, and preferences. Nine pregnant women recruited in a pregnancy clinic discussed their thoughts about pregnancy and the meaning of this study. The patients were not involved in the recruitment to and conduct of the study. They were given the option to request the results be disseminated to them.

## Results

### The Structure of Perceptions of Pregnancy

The clustering structure (presented in Supplementary Material 1) represented the proximity relations between the items adequately: *R*^2^ = 0.65, Stress Formula = 0.08. The configuration discovered revealed three major clusters: (1) evaluative aspects of pregnancy; (2) physiological-medical aspects of pregnancy; and (3) future considerations. The major evaluative cluster was divided into four sub-clusters: (a) motivations to conceive (e.g., social, religious, or biological motivations); (b) ambivalence about pregnancy (expressed by negative feelings like guilt, confusion); (c) social aspects of pregnancy (giving help to pregnant women, etc.); and (d) positive emotions (e.g., pride and excitement). The second major cluster, physiological-medical aspects, included two sub-clusters: (a) risks and medical examinations (e.g., thoughts about doctors and hospitals, risk for the woman, and delivery complications); and (b) sensations and symptoms (e.g., dizziness and pain). The third major cluster, thoughts about future, included two sub-clusters: (a) personal change; and (b) parenthood and family change. Items composing sub-clusters were averaged as scale scores. Five items were eliminated due to inappropriate psychometric properties (e.g., zero correlation with other items), and two other items were eliminated due to redundancy. The final version of the questionnaire used in subsequent analyses included 62 items (see Supplementary Material 2). The means, standard deviations, and reliabilities of the scale scores are presented in Table 2.

**Table 2:** Means, standard deviations, and reliabilities of the scale scores.

Scale	Number of items	*N* = 290		
		*M*	SD	*Α*
**Evaluative**	**27**	**3.65**	**0.85**	**0.87**
Motivations	3	2.37	1.29	0.57
Ambivalence	7	2.32	0.91	0.58
Social	5	3.47	1.26	0.72
Positive emotions	12	4.84	1.27	0.89
**Physiological health**	**18**	**4.21**	**1.11**	**0.89**
Risks and check-ups	11	4.35	1.20	0.8711
Symptoms and sensations	7	3.87	1.25	0.81
**Thoughts about future**	**17**	**4.36**	**0.98**	**0.83**
Personal change	8	3.98	1.11	0.70
Parenthood	9	4.81	1.20	0.81

Pearson correlations between the questionnaire scales and demographic variables revealed only the following significant findings: age was negatively associated with evaluative aspects of pregnancy (*r* = −0.17, *p* < 0.01), particularly with motivation to conceive (*r* = −13, *p* < 0.05), ambivalence (*r* = −0.16, *p* < 0.01) and positive emotions (r = 0.12, *p* < 0.05); participants’ age was also associated with thoughts about physiological symptoms (*r* = −0.15, *p* < 0.05) and with thoughts about parenthood (*r* = −0.22, *p* < 0.01). Education was negatively correlated with the evaluative scale (*r* = −0.13, *p* < 0.05), but not with any particular sub-scale that composes it. Education was significantly correlated with thoughts about risks and medical examinations (*r* = −0.16, *p* < 0.01) and with thoughts about physical symptoms (*r* = −14, *p* < 0.05). Table 3 presents moderate significant correlations among most sub-scale scores.

**Table 3:** Correlations among the pregnancy perception questionnaire scale. *N* = 290.

Scale	P1	P2	P3	P4	**PH**	PH1	PH2	**F**	F1	F2
**P Evaluative**	**0.68**	**0.55**	**0.81**	**0.70**	**0.45**	**0.37**	**0.42**	**0.49**	**0.32**	**0.53**
P1 Motivations		0.28	0.36	0.24	**0.21**	0.17	0.20	**0.22**	0.18	0.20
P2 Ambivalence			0.35	0.20	**0.28**	0.16	0.35	**0.37**	0.36	0.29
P3 Social				0.53	**0.46**	0.39	0.42	**0.46**	0.32	0.49
P4 Positive Emotions					**0.32**	0.30	0.29	**0.36**	0.10	0.52
**PH Physiological**						**0.86**	**0.90**	**0.56**	**0.45**	**0.53**
PH1 Risks							0.56	**0.43**	0.32	0.44
PH2 Symptoms								**0.54**	0.45	0.49
**F Future**									**0.85**	**0.87**
F1 Personal Change										0.50
F2 Parenthood										

*All correlations except the correlation between positive emotions and thoughts about personal change are significant (*p* < 0.01)*.

Independent sample t-tests were performed to compare pregnancy perceptions of pregnant versus non-pregnant participants (Table 4), and men versus women (Table 5). As can be seen in Table 4, compared to non-pregnant participants, pregnant participants associated pregnancy with less thought about parenthood and more thought about medical aspects and risks associated with pregnancy. Non-pregnant participants expressed more ambivalence about pregnancy compared to pregnant participants. Women experienced more pregnancy-related positive emotions than men (Table 5) and also thought more about symptoms and sensations. Compared to men, women also thought more about future issues. Pregnant women felt less ambivalence toward pregnancy compared to non-pregnant women, thought more about risks and medical examinations, but less about parenthood. Multivariate Analysis of Variance of pregnancy perceptions by gender and pregnancy condition revealed two main effects for gender and being pregnant, with no interaction effect (as presented in Tables 4, 5). Repeating the analyses while controlling the effects of age and education (as co-variates) nullified the main effects of pregnancy on medical examinations and gender on ambivalence.

**Table 4:** Means, standard deviations, and F-scores of the scale scores for pregnant and non-pregnant participants.

Scale	Number of items	Pregnant *N* = 91		Non-pregnant *N* = 198		
		*M*	SD	*M*	SD	*F*
**Evaluative**	**27**	**3.09**	**0.84**	**3.31**	**0.85**	**6.61**
Motivations	3	2.08	1.25	2.51	1.33	7.86^**^
Ambivalence	7	2.06	0.79	2.43	0.88	11.11^**^
Social	5	3.46	1.31	3.47	1.32	0.25
Positive emotions	12	4.76	1.26	4.84	1.27	1.43
**Physiological health**	**18**	**4.23**	**1.01**	**4.04**	**1.19**	**0.98**
Risks & Medicine	11	4.52	1.11	4.19	1.27	3.17
Symptoms & sensations	7	3.95	1.37	3.90	0.98	0.00
**Thoughts about future**	**17**	**4.13**	**1.00**	**4.47**	**1.05**	**9.24** ^**^
Personal change	8	3.39	1.05	4.00	1.13	3.13
Parenthood	9	4.47	1.22	4.95	1.15	11.82^**^

** *p < 0.05*.

**Table 5:** Means, standard deviations, and F-scores of the scale scores for men and non-women participants.

Scale	Number of items	Men *N* = 80		Women *N* = 209		
		*M*	SD	*M*	SD	*F*
**Evaluative**	**27**	**3.14**	**0.91**	**3.28**	**0.83**	**3.12**
Motivations	3	2.48	2.42	2.33	1.29	0.27
Ambivalence	7	2.32	0.92	2.31	0.86	0.03
Social	5	3.34	1.20	3.51	1.36	1.68
Positive emotions	12	4.4	1.13	5.0	1.20	14.79^**^
**Physiological health**	**18**	**3.68**	**1.13**	**4.26**	**1.11**	**13.87** ^**^
Risks & Medicine	11	4.20	1.22	4.32	1.24	0.71
Symptoms	7	3.17	1.29	4.20	1.24	33.39^**^
**Thoughts ab. future**	**17**	**4.05**	**0.95**	**4.48**	**1.00**	**12.51** ^**^
Personal change	8	3.64	1.08	4.05	1.10	8.95^**^
Parenthood	9	4.47	1.23	4.92	1.16	9.74^**^

** *p < 0.05*.

## Discussion

Our study has examined the phenomenological concept of pregnancy. The results indicate that the cognitive schema of pregnancy is multi-leveled and composed of three major clusters (or categories): “evaluative,” “physiological-medical,” and “thoughts about future.” The “evaluative” cluster included four sub-clusters: “motivations to conceive” (e.g., social, religious, or biological motivations), “ambivalence” (e.g., guilt and confusion), “social aspects” (e.g., help or positive social attitude toward pregnant women), and “positive emotions” (e.g., proud and excitement). The second major cluster “physiological-medical” included two sub-clusters: “risk and medical examinations” (e.g., thoughts about doctors and hospitals and risk to the woman) and “sensations and symptoms” (e.g., dizziness and pain). The third major cluster, “thoughts about the future,” included two sub-clusters: “personal change” and “parenthood and family change.”

It is interesting to note that thoughts about delivery were represented in the cluster of “symptoms and sensations” rather than in “risks and medical examinations.” It seems that compared to delivery, pregnancy is a much broader cognitive construct, in which delivery is only one component. A recent study focusing on perceptions of birth *per se* found that birth perceptions are composed of two factors: beliefs about birth as a natural process and beliefs about birth as a medical process (Gati et al., 1995; Preis and Benyamini, 2017). However, as indicated by our findings, when focusing on pregnancy, perceived as replete with medical risks and examinations, delivery seems to be represented by its natural process facet, as relatively less risky, although painful and physically inconvenient. This attests to the general feelings of safety concerning delivery in the modern world.

Our participants’ general perception of pregnancy was positive, evoking positive emotions (e.g., self-fulfillment, excitement, and pride). This is consistent with theoretical propositions (Enkin, 1994; Beck and Beck-Gernsheim, 1995) that unlike the past, most of today’s pregnancies are planned, therefore people expect more advantages than costs from having children. Our results also support the notion that given the increases in quality of life and individualization, parents do not expect practical, material, or economic benefits from having children. The real perceived reward of having children is emotional (Enkin, 1994). Positive pregnancy-related emotions were indeed found correlated with social aspects of pregnancy (e.g., spouse support, positive evaluation of others, and help), and with thoughts about parenthood (e.g., passing values, functioning as a parent, and responsibility). These findings suggest the existence of reciprocal augmenting influences between psychological and social factors in a society that cherishes family and children.

According to Beck and Beck-Gernsheim (Beck and Beck-Gernsheim, 1995), modern parenthood has become an increasingly responsible task. The list of requirements from a parent is long, including provision of a private room and pocket money, toys, and sport activities. More than ever before, people deliberate on whether or not they are emotionally and economically fit to bear a child. Our results support this notion. The scale composed of thoughts about parenthood (financial considerations, spouse relation stability, etc.) scored relatively high. Urdze and Rerrich (1981) found that thoughts like these typically involve feelings of insecurity, ambivalence, and other contradictions. Accordingly, the “ambivalence” scale was found correlated with “thoughts about the future,” although this correlation was low.

The introduction of new technologies for detecting fetus abnormality has resulted in increasing perceptions of pregnancy-related risks and anxieties (Istvan, 1986). Our results support these prior observations. The “risk and medical examinations” scale scored high and correlated significantly with the “evaluative” score, especially with “social aspects of pregnancy.” This is consistent with findings about the interplay between higher valued goals and stress generation processes (Auerbach et al., 2011). It is possible that highly positive evaluations of pregnancy direct more attention to risks associated with it. Another possible explanation for the association between “risk and medical examinations” and evaluative “social aspects of pregnancy” is the increased availability of social support from accompanying partners, doctors, and other pregnant couples being met in clinics when attending prenatal examinations.

### Cross Groups’ Comparisons

The comparisons between pregnant and non-pregnant participants revealed that for pregnant participants, pregnancy was associated with significantly *less* thoughts about parenthood and *more* thoughts about risk contents. These findings are predicted by the Construal Level Theory (Liberman and Trope, 2008). Non-pregnant participants, who perceived pregnancy as a future event, represented it by high level construals (i.e., general thoughts about parenthood), while pregnant participants, for whom pregnancy is an ongoing situation, represented it by low-level concrete features (such as pregnancy risks). The finding that non-pregnant participants scored higher on “reasons to conceive” and “ambivalence” (high level construals) further support the model. In a planned-pregnancy society, pregnant women are no longer ambivalent and no longer think about justifications for conceiving (Beck and Beck-Gernsheim, 1995).

Women were found to perceive more positive emotions about pregnancy than men. This is in line with evolutionary psychology (Buss, 1995), suggesting that compared to men, women’s success in passing their genes to future generations depends on their investment in a finite number of children they can give birth to and rear. Therefore, evolutionarily, it was more important for women to develop positive emotional rewards from pregnancy and child rearing. Another possible explanation is that men are more reluctant than women to report on specific emotions (Simon and Nath, 2004). Women were also found to think more than men about symptoms and sensations and about future implications (i.e., effects on one’s career, economic aspects, and stability of relationship), most likely because pregnancy has more impacts on these issues for them.

## Implications

As illustrated in the present research, pregnancy perceptions are useful indicators for testing theoretical predictions derived from Evolutionary Psychology (Buss, 1995), Modernization Theory (Beck and Beck-Gernsheim, 1995), and Construal Level Theory (Liberman and Trope, 2008). It would be especially interesting to investigate relations between pregnancy perceptions and reproduction-related behaviors, such as family-planning practices, utilization of pregnancy screening services, and family size.

Caretakers of pregnant couples and patients in fertility clinics may also find that familiarizing themselves with the constructs of pregnancy perceptions is useful for improving client-centered communication and treatment. Using the *Pregnancy Perceptions Questionnaire* in clinical practice may help identify specific problem areas, such as ambivalence, excessive risk perceptions, or limited social support that need attention. A recent study (Jessop et al., 2014) reported that representations of pregnancy accounted for up to 30% and 39% of the variance in physical and mental health, respectively, among women in the last trimester of pregnancy. Pregnancy representations were measured in that study by an adaptation to pregnancy of the Revised Illness Perception Questionnaire (Moss-Morris et al., 2002). We expect that due to its specificity, the *Pregnancy Perceptions Questionnaire* would potentially better predict physical and mental health of pregnant women even better (which can also be used to develop psychological interventions for supporting such women and their carers; Ditmitrov and Vazova, 2019).

## Strengths and Limitations of This Study

- Caretakers of pregnant couples and patients in fertility clinics may also find that familiarizing themselves with the constructs of pregnancy perceptions is useful for improving patient-centered communication and treatment.
- Representations of pregnancy (usually measured by an adaptation of the Revised Illness Perception Questionnaire) accounted for up to 30% and 39% of the variance in physical and mental health, respectively, among women in the last trimester of pregnancy.
- We expect that due to its specificity, our proposed *Pregnancy Perceptions Questionnaire* will enable better predicting physical and mental health of pregnant women in clinical practice, and will also help identify specific problem areas, such as ambivalence, excessive risk perceptions, or limited social support that need attention.

Additional research is needed to confirm the current structure of pregnancy perception in larger and varied populations. Given the recognized socio-cultural impacts on all aspects of childbearing (Brislim, 1983; Jordan, 1992; Shiloh et al., 1993), it is impossible to generalize from one study conducted within one country to universal meanings. Future cross-cultural comparisons using the pregnancy perception questionnaire may enable separating the universal from the local in the contents and structure of pregnancy perception. A case in point is evidence of changes in immigrants’ perceptions. After the demise of the former Soviet Union, more than 700,000 immigrants have resettled in Israel in the 1990s. Among the many adaptations required by immigration, there is evidence of changes in family-planning attitudes, knowledge, and practices (Remennick I., 1999). Further research using our questionnaire may compare pregnancy perceptions between Russian Jews who live in Russia, immigrants from the former Soviet Union to Israel, and native Israelis (Remennick, 1998). Such research may provide new insights into universal and cultural sources of pregnancy perceptions.

We focused on comparing perceptions of pregnant versus non-pregnant participants and men versus women, basing our hypotheses on theoretical grounds (Evolutionary Psychology, Modernization Theory, and Temporal Construal Theory). Other comparisons may help elucidate additional aspects of pregnancy perceptions. For example, future research may focus on the effects of infertility experience on pregnancy perceptions by comparing perceptions among couples who have become pregnant easily versus those who experienced infertility. Case reports and qualitative research suggest that previously infertile women are prone to experience an extremely “tentative” pregnancy, characterized by anxiety about pregnancy outcome, depression, and denial of physiological symptoms (Sandelowski, 1987; Burns, 1996; Mcmahon et al., 1999). It would be interesting and important to extend this research field by investigating the effects of infertility on pregnancy perceptions. Goal-attainment theories may guide predictions of such research (Feldman et al., 2009).

Our sample size was adequate but limited our ability to examine sub-samples with differing demographic backgrounds. Investigating pregnancy perceptions in larger samples would enable a more thorough examination of age, education, social class, previous pregnancies, and number of children influences on pregnancy perceptions. Finally, our study measured pregnancy perceptions on one occasion. It is desired to develop longitudinal studies with repeated measurement points to understand how perceptions of pregnancy develop and change over time and life experiences, including during pregnancy.

## Data Availability Statement

The raw data supporting the conclusions of this article will be made available by the authors, without undue reservation.

## Ethics Statement

The studies involving human participants were reviewed and approved by the Tel Aviv University Ethics Committee. The patients/participants provided their written informed consent to participate in this study.

## Author Contributions

LB designed and conducted the study, analyzed the data, and prepared the manuscript. SS contributed to the design of the study, interpretation of the results, and writing of the manuscript. LA contributed to the analysis and interpretation of data for the work and drafting the work and revising it critically for important intellectual content. IV contributed to the analysis and interpretation of the results and the writing up of the manuscript. All authors contributed to the article and approved the submitted version.

## Conflict of Interest

The authors declare that the research was conducted in the absence of any commercial or financial relationships that could be construed as a potential conflict of interest.
