# Supplementary material for: The Latent Perception of Pregnancy
Source: Front Psychol. 2022 Mar 24;13:589911. doi: 10.3389/fpsyg.2022.589911 (PMC8987224; doi:10.3389/fpsyg.2022.589911)
Supplement: Supplementary file 1 [file Table_1.docx]

**Supplementary Material 1**

**Statistical Analysis Protocol**

A clustering algorithm called ADDTREE (i.e., additive similarity tree, Sattath & Tversky, 1977) was performed using a similarity matrix (Pearson correlations) among the questionnaire items (see below). This procedure was aimed to group the items into internally consistent clusters. ADDTREE is generally used to provide a clustering representation of a concept and represent dissimilarities among items. This method has been successfully used to analyze the structure of career related aspects (Preis & Benyamini, 2017), as well as health behaviors and illness attributions (Shiloh et.al, 2002). ADDTREE is especially attractive because it graphically represents the proximity matrix in the form of an additive or ‘path length’ tree, in which the variables are divided into clusters and sub-clusters according to the proximity between them (based on the correlation matrix). The distance between any pair of items is represented in the clustering structure by the sum of horizontal arcs on the shortest path connecting them. Items that have common features are clustered together, while different clusters represent distinctive features. In our study, the relationship between an item and the corresponding cluster is essentially the relationship between a specific statement in the questionnaire and the notion of pregnancy represented by a set of statements included in the same cluster. The accuracy of the analysis was measured by 2 goodness of fit indices: Kruskal’s Stress formula, which is an index of the stability of the ADDTREE solution and ranges from zero (perfectly stable) to 1 (perfectly unstable), and R2 (the linear variance accounting for each solution). The larger the R2, the better the configuration represents the data.

Two independent researchers reached agreement about labeling the clusters according to their constituent items. Items that composed each sub-cluster were averaged to form sub-scale scores. The psychometric properties of the questionnaire were examined, focusing on scales’ means, standard deviations and internal reliabilities (Cronbach’s alpha). Finally, using t-tests for independent samples, scale scores were compared between men and women, and between pregnant and non-pregnant participants.

A clustering algorithm ADDTREE (additive similarity tree, Sattath & Tversky, 1977) using a similarity matrix (Pearson correlations) among the questionnaire items

peer pressure

Coddling the woman

guilt

pregnancy is like a disease

strangeness

embarrassment

feeling of ownership

egoistic motivation

religious motivation

biological motivation

(in)confidence in father's identity

inclusion of the significant others and its reactions

positive evaluation from the environment

giving and needing help

pregnancy is a sign of health

receiving attention from partner

Superstitious beliefs

surprise

baby dreams

building a nest

maternal instinct

"the fruit of love"

curiosity/expectation

pleasure

Inner peace

excitement

astonishment from the wonder

confidence

pride

self-actualization

fear/worry

limitations in a woman's life

fear for a woman's health

Endangerment of a woman

labor complications

danger to the fetus

The need to protect the wife

The need to protect the fetus

thoughts about doctors

fear for the fetus' safety

medical checkups for the woman

medical checkups for the fetus

mood swings

taste changes and morning nauseas

physical changes to appearance

changes and physical development

thoughts about pain and weakness

fears about labor

thoughts about discomfort

changes in family relations

thoughts about damage to career

what should we do before the baby is born?

thoughts about task distribution

thoughts about loss of freedom

ambivalence

feeling of uncertainty

what will happen after the baby's born?

Feeling of change

drastic life changes

Thoughts about the future

feeling of responsibility

thoughts about parental function

Responsibility for the fetus' safety

thoughts about the world where the baby is born

thoughts about values and education

thoughts about economic sacrifices

thoughts about the stability of the relationship

preparations for labor

References

Preis, H., & Benyamini, Y. (2017). The birth beliefs scale - a new measure to assess basic beliefs about birth. Journal of Psychosomatic Obstetrics & Gynecology, 38, 73-80.

Sattath, S., & Tversky, A. (1977). Additive similarity trees. Psychometrika, 42, 319-154.

Shiloh, S., Rashuk-Rosenthal, D. and Benyamini, Y. (2002). Illness attributions: their structure and associations with other illness cognitions and perceptions of control. Journal of Behavioral Medicine, 25, 373-394.
