# Supplementary material for: The Latent Perception of Pregnancy
Source: Front Psychol. 2022 Mar 24;13:589911. doi: 10.3389/fpsyg.2022.589911 (PMC8987224; doi:10.3389/fpsyg.2022.589911)
Supplement: Supplementary file 2 [file Table_2.docx]

**Supplementary Material 2**

**The Pregnancy Perceptions Questionnaire**

**Think about pregnancy for a minute**

Pregnancy makes you think and feel different things. Here’s a list of thoughts you might have when thinking about pregnancy (yours or someone else’s). Check near each one how much it fits your thoughts about pregnancy.

| **Perfectly describes my thoughts** |  |  |  |  |  | **Didn’t even consider it** |
| --- | --- | --- | --- | --- | --- | --- |
| **7** | **6** | **5** | **4** | **3** | **2** | **1** |
|  |  |  |  |  |  |  |

1. ___ thoughts about limitations and difficulty in the woman’s life
2. ___ fear for the woman’s health because of the pregnancy/labor
3. ___ thoughts about changes in family relations (siblings, parents…)
4. ___ thoughts about task distribution
5. ___ feelings of fear/worry
6. ___ feelings of pleasure/happiness/dreams coming true
7. ___ thoughts about egoistic motives for having a child
8. ___ thoughts about taste changes and morning nauseas
9. ___ baby dreams/imagining the baby
10. ___ feelings of embarrassment/confusion
11. ___ thoughts about sharing the environment the news and social reactions
12. ___ thoughts about social pressure to get pregnant
13. ___ thoughts about the biological need to reproduce and pass the genes
14. ___ thoughts about confidence about the father’s identity
15. ___ feeling of ownership over the baby
16. ___ thoughts about “building a nest” for the baby and preparing for him
17. ___ thoughts about positive evaluation from the environment
18. ___ feelings of seriousness/responsibility
19. *thoughts about maternal/paternal instinct (deleted)*
20. ___ ambivalence
21. ___ thoughts about physical changes in appearance
22. ___ feeling of life change

| **Perfectly describes my thoughts** |  |  |  |  |  | **Didn’t even consider it** |
| --- | --- | --- | --- | --- | --- | --- |
| **7** | **6** | **5** | **4** | **3** | **2** | **1** |
|  |  |  |  |  |  |  |

1. ___ thoughts about damage to career
2. ___ thoughts about need to protect the woman and be careful
3. ___ pregnancy is dangerous to the fetus
4. ___ feeling of curiosity/expectation
5. *___ thoughts about “the fruit of love”, the fetus as a part of the partner (deleted)*
6. ___ thoughts about what you should do before the baby is born (chores, travel…)
7. ___ thoughts about medical checkups for the woman
8. ___ thoughts about getting attention from the spouse
9. *___ thoughts about pregnancy as a sign for health (deleted)*
10. ___ thoughts about changes and development
11. ___ thoughts about doctors, hospitals, etc.
12. ___ thoughts about labor complications
13. ___ pregnancy is dangerous for the woman
14. ___ need to act in favor of the fetus (diet, drinking, smoking)
15. ___ thoughts about mood changes
16. ___ thoughts about excitement
17. ___ feelings of calmness/inner peace/gentleness
18. ___ thoughts about a foreign object inside
19. ___ thoughts about the future
20. ___ thoughts about religious commandments
21. ___ thoughts about celebrating the woman – the woman is the most important
22. ___ thoughts about needing and giving help from and to the environment
23. ___ feeling of confidence/power
24. ___ the miracle of childbirth
25. ___ fear for the fetus’ health

| **Perfectly describes my thoughts** |  |  |  |  |  | **Didn’t even consider it** |
| --- | --- | --- | --- | --- | --- | --- |
| **7** | **6** | **5** | **4** | **3** | **2** | **1** |
|  |  |  |  |  |  |  |

1. ___ thoughts about the world where the baby is born
2. ___ thoughts about passing values to the next generation
3. ___ thoughts about drastic changes in life
4. ___ thoughts about self-actualization
5. ___ thoughts about pain/weakness
6. *Superstitions (deleted)*
7. *feeling of surprise (deleted)*
8. ___ thoughts about life after the birth (depression, functioning)
9. ___ feeling of pride/creating life
10. ___ feeling of loss of freedom for the baby
11. ___ thoughts of functioning as parents
12. ___ feeling of seriousness/responsibility: the baby needs the woman
13. ___ uncertainty
14. ___ pregnancy is like a disease
15. ___ fears of labor itself (pain, etc.)
16. *___ guilt (deleted)*
17. ___ thoughts about physical discomfort
18. ___ thoughts about economic sacrifices
19. ___ thoughts about relationship stability
20. ___ thoughts about actual preparations for the labor
21. ___ thoughts about medical checkups for the fetus
