# Supplementary material for: The Latent Perception of Pregnancy
Source: Front Psychol. 2022 Mar 24;13:589911. doi: 10.3389/fpsyg.2022.589911 (PMC8987224; doi:10.3389/fpsyg.2022.589911)
Supplement: Supplementary file 3 [file Table_3.DOCX]

**Supplementary Material 3**

**Limitations and desired future studies**

Additional research is needed to confirm the current structure of pregnancy perception in larger and varied populations. Given the recognized socio-cultural impacts on all aspects of childbearing [40-42], it is impossible to generalize from one study conducted within one country to universal meanings. Future cross-cultural comparisons using the pregnancy perception questionnaire may enable separating the universal from the local in the contents and structure of pregnancy perception. A case in point is evidence of changes in immigrants’ perceptions. After the demise of the former Soviet Union, more than 700,000 immigrants have resettled in Israel in the 1990s. Among the many adaptations required by immigration, there is evidence of changes in family-planning attitudes, knowledge and practices [43]. Further research using our questionnaire may compare pregnancy perceptions between Russian Jews who live in Russia, immigrants from the former Soviet Union to Israel and native Israelis [44]. Such a research may provide new insights into universal and cultural sources of pregnancy perceptions.

We focused on comparing perceptions of pregnant versus non-pregnant participants and men versus women, basing our hypotheses on theoretical grounds (Evolutionary Psychology, Modernization Theory, Temporal Construal Theory). Other comparisons may help elucidate additional aspects of pregnancy perceptions. For example, future research may focus on the effects of infertility experience on pregnancy perceptions by comparing perceptions among couples who have become pregnant easily versus those who experienced infertility. Case reports and qualitative research suggest that previously infertile women are prone to experience an extremely “tentative” pregnancy, characterized by anxiety about pregnancy outcome, depression, and denial of physiological symptoms (45-47). It would be interesting and important to extend this research field by investigating the effects of infertility on pregnancy perceptions. Goal-attainment theories may guide predictions of such research [48].

Our sample size was adequate, but limited our ability to examine sub-samples with differing demographic backgrounds. Investigating pregnancy perceptions in larger samples would enable a more thorough examination of age, education, social class, previous pregnancies, and number of children influences on pregnancy perceptions. Finally, our study measured pregnancy perceptions on one occasion. It is desired to develop longitudinal studies with repeated measurement points to understand how perceptions of pregnancy develop and change over time and life experiences, including during pregnancy.
